# Own-Synthetize Nanoparticles to Develop Nano-Enhanced Phase Change Materials (NEPCM) to Improve the Energy Efficiency in Buildings

**DOI:** 10.3390/molecules24071232

**Published:** 2019-03-29

**Authors:** Camila Barreneche, Marc Martín, Jaume Calvo-de la Rosa, Marc Majó, A. Inés Fernández

**Affiliations:** 1 Department of Materials Science and Physical Chemistry, Universitat de Barcelona, Martí i Franqués 1–11, 08007 Barcelona, Spain; marcml451@gmail.com (M.M.); jaumecalvo@ub.edu (J.C.-d.l.R.); mmajorob7@alumnes.ub.edu (M.M.); 2 Birmingham Centre for Energy Storage & School of Chemical Engineering, University of Birmingham, Birmingham B15 2TT, UK

**Keywords:** nano-enhanced phase change materials (NEPCM), thermal energy storage (TES), energy efficiency, buildings, fatty acids, nanofluid, DSC, hot wire

## Abstract

The use of adequate thermal energy storage (TES) systems is an opportunity to increase energy efficiency in the building sector, and so decrease both commercial and residential energy consumptions. Nano-enhanced phase change materials (NEPCM) have attracted attention to address one of the crucial barriers (i.e. low thermal conductivity) to the adoption of phase change materials (PCM) in this sector. In the present study two PCM based on fatty acids, capric and palmitic acid, were nano-enhanced with low contents (1.0 wt.%, 1.5 wt.% and 3.0 wt.%) of copper (II) oxide (CuO) nanoparticles. Copper (II) oxide (CuO) was synthesized via coprecipitation method obtaining 60–120 nm diameter sized nanoparticles. Thermal stability and high thermal conductivity were observed for the nano-enhanced phase change materials (NEPCM) obtained. Experimental results revealed remarkable increments in NEPCM thermal conductivity, for instance palmitic acid thermal conductivity was increased up to 60% with the addition of 3 wt.% CuO nanoparticles. Moreover, CuO nanoparticles sedimentation velocity decreases when increasing its content.

## 1. Introduction

Nowadays, humankind faces a global and unavoidable energy challenge. First, world populations are still growing at unprecedented rates: in the twentieth century the increase in world population was three times greater than during the entire previous history of humanity. Moreover, as it is comprehensible, hundreds of millions of people want a higher standard of living which comes with an incredibly high associated energy cost: Global energy demand grew by 2.1% in 2017 [1]. Secondly, climate change, particularly the global temperature rise driven by greenhouse gases, such as CO_2_. These points draw an unsustainable future in terms of energy. In this regard, renewable energy and energy efficiency have been hot topics in recent years.

Buildings energy efficiency has attracted attention due to the high energy consumption, as the building sector accounts for the 36% of energy consumption worldwide [2]. In particular, heating, ventilation, and air conditioning (HVAC) systems are the major contributor (50%) of the buildings energy consumption [3]. Thermal energy storage (TES) systems and latent heat energy storage (LHES) are able to improve HVAC systems energy efficiency, hence reducing greenhouse gas emissions. These types of systems are simply contained mediums which are able to charge thermal energy and release it in a controlled way. Thus, phase change materials (PCM) can reduce the temperature fluctuations in a room, therefore enabling a more dynamic use of energy. Nevertheless, due to the high cost of these technologies, it is crucial to apply adequate control strategies to improve their overall performance and make them cost-effective [4]. In addition, LHES systems have been applied to building integrated photovoltaics (BIPV) to absorb heat to control temperature rise and improve the systems efficiency [5]. Recently in this regard, Sun et al. [6] studied binary hydrated salts to be applied in solar water heating (SWH) systems.

This study is based on the materials which are able to store latent heat that are known as PCM. When a heat source is applied, the solid materials will increase their temperature until reaching the melting point where the phase change will take place. For this change of state, the materials need a certain amount of energy and during this process the heat supplied will not increase the temperature of the material until the phase change is completely given. During this process, the amount of heat absorbed is known as melting enthalpy (ΔH_m_) and it is defined as latent heat.

Although the main application of this study is the use of PCM in buildings, there are many other uses for these materials, such as the following: thermal storing of solar energy [7], heating systems [8], integrated in clothes [9], food transport refrigeration [10], thermal protection in alimentation [11], and thermal comfort in vehicles [12].

Fatty acids have received much attention to be used as PCM in building applications [13]. However, it is worthwhile to point out these phase change materials have disadvantages that limit their application. One of the main limitations of the use of fatty acids as PCM are their poor thermal conductivity, which might slow the heat exchange during storage and release processes. Nanomaterials are ruled by quantum mechanics in the small dimensions but are able to show impressive properties at a macroscopic scale. In this way, nanomaterials demonstrate a wide variety of potential applications in many fields. The energy efficiency in the building sector is no exception and research studies in recent years have shown the high potential of nano-enhanced PCM in TES systems for this sector [14]. The dispersion of high thermal conductive nm-sized structures, such as nanoparticles, in a PCM base fluid can significantly increase its thermal conductivity and so improve its capabilities during storage and release processes. In this regard, various carbon base nanomaterials have been used in recent years. Şahan et al. [15] reported that the addition of 1.0 wt.% multi-walled carbon nanotubes (MWCNTs) to paraffin PCM significantly increased thermal conductivity (40%) and latent heat (10%). Yo et al. [16] developed fatty acid based PCM with carbon nanotubes (CNTs) and exfoliated graphite nanoplatelets (xGnP). The nanocomposite with 3 wt.% xGnP showed a remarkable thermal conductivity increase (297%) while CNT-based PCM achieved an increase of 218% for the same mass fraction content. Furthermore, inorganic nanostructures, such as metal and metal oxide nanoparticles, were proved to provide valuable thermal conductivity enhancement in a cost-effective manner. For instance, Wang et al. [17] studied the addition of different amounts (1.0 wt.%, 2.0 wt.%, 3.0 wt.%, 4.0 wt.% and 5.0 wt.%) of Fe_2_O_3_ nanoparticle into paraffin. Results showed an increased trend of thermal conductivity with Fe_2_O_3_ nanoparticle content, except for the nanocomposite with 3.0 wt.% Fe_2_O_3_, which thermal conductivity enhancement was the highest (30%).

Nevertheless, it is important to highlight that nm-sized materials may have a toxic effect. So, an appropriate approach must be taken to integrate nano-enhanced PCM into buildings.

The main idea of this study is a complete characterization of the new nano-enhanced phase change materials (NEPCM) with a maximum nanoparticle dispersion in a base fluid (liquid-PCM). The high interest concerning NEPCM is due to the unexpected improvement of thermal conductivity within the dispersion being obtained by adding a small percentage of nanoparticles [18] even though other researchers have measured the expected enhancement of thermal conductivity by following the mixtures, law of Maxwell was also observed [19]. 

## 2. Results

### 2.1. Nanoparticles Characterization

**Figure 1:**
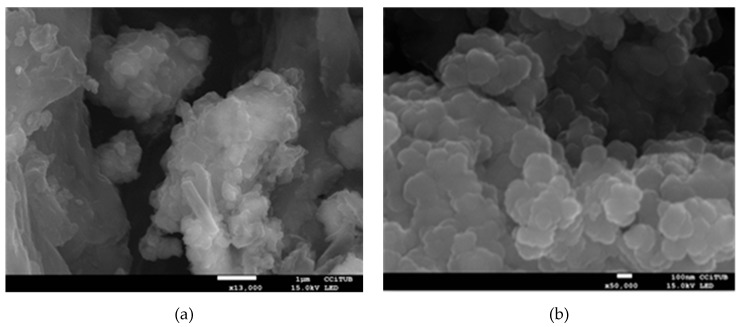
SEM images of the CuO nanoparticles added to the manufactured NEPCM at (**a**) 13,000× magnification AND (**b**) 50,000× magnification.

**Sample size**: SEM images are shown in Figure 1 where the nanoparticles size was measured. The nanoparticles diameters are around 60–120 nm as is shown in Figure 1b). To determine the sedimentation velocity, a mean nanoparticle size value of 90 nm will be used.

**Figure 2:**
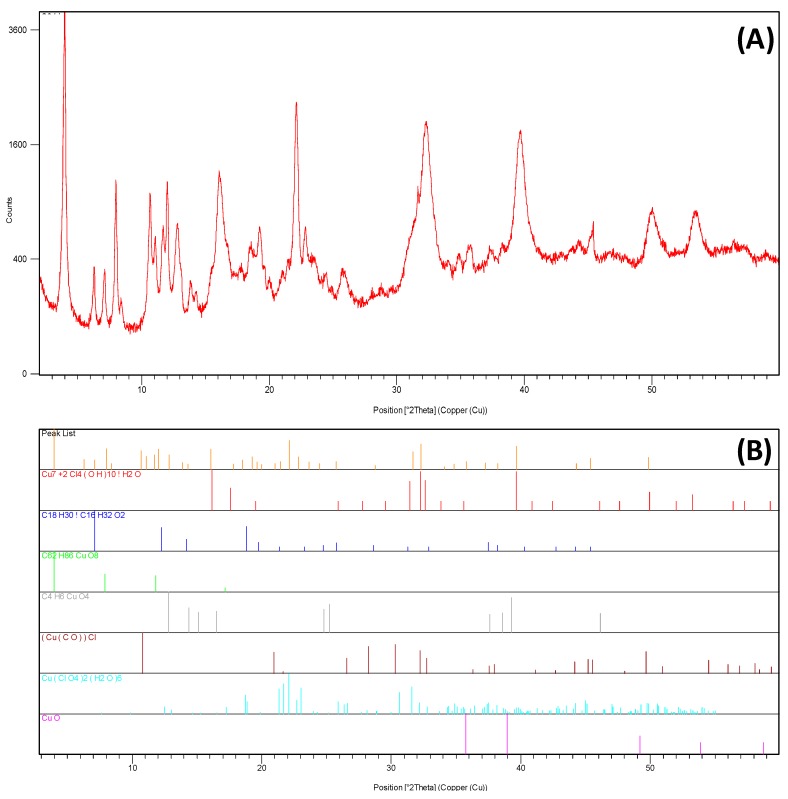
(**A**) XRD pattern of the obtained product, and (**B**) identification patterns of the different species matched.

**Composition:** The x-ray diffraction pattern depicted in Figure 2 shows a high amorphous content based on the pronounced non-linear background. Possibly it is the result of having used octanoic acid as surfactant. The desired phase (tenorite, CuO) is identified in the diffractogram, but the pattern is really complex due to the presence of a high concentration of other compounds. They basically consist on a copper oxide or chloride linked with organic or hydrated structures, forming Cu_7_+2Cl_4_(OH)_10_·H_2_O, C_62_H_86_CuO_8_, C_4_H_6_CuO_4_, (Cu(CO))Cl and Cu(ClO_4_)_2_·6(H_2_O). The first conclusion that should be taken from these results is that the oxidation process has not been completed because chloride compounds are still present in the sample. Then, it is seen that the ions that are initially dissolved in the water and octanoic acid solution can react with the octanoic acid or get hydrated and form these complex structures. Possibly, the formation of these compounds is the reason why the oxidation process becomes harder to be completed.

### 2.2. NEPCM Characterization

**Figure 3:**
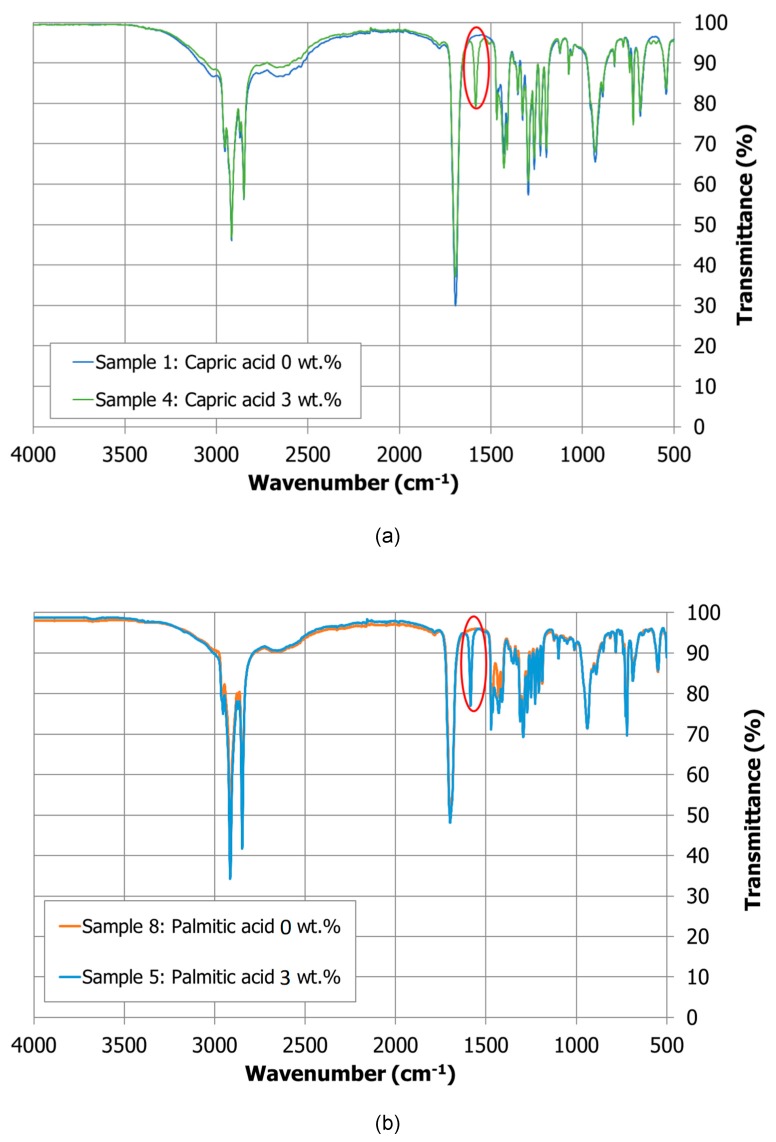
FT-IR spectra of capric acid (**a**) and palmitic acid (**b**) samples under study.

**Composition:** By comparing the FT-IR spectra obtained from the sample with and without nanoparticles allows understanding the formation of any new substance, if the original peaks are preserved or are changed. A new peak appears around 1580 cm^−1^ in the NEPCM samples that is not shown in the fatty acid without nanoparticles. The Figure 3 shows this extra peak for the capric and palmitic acids as an example. Notice that it is the only clear changed registered in the spectra. While the elucidation of this peak is not clear, a possible explanation may be that the copper oxide promotes the formation of carboxylate groups that show a characteristic peak in this region [20]. 

**Figure 4:**
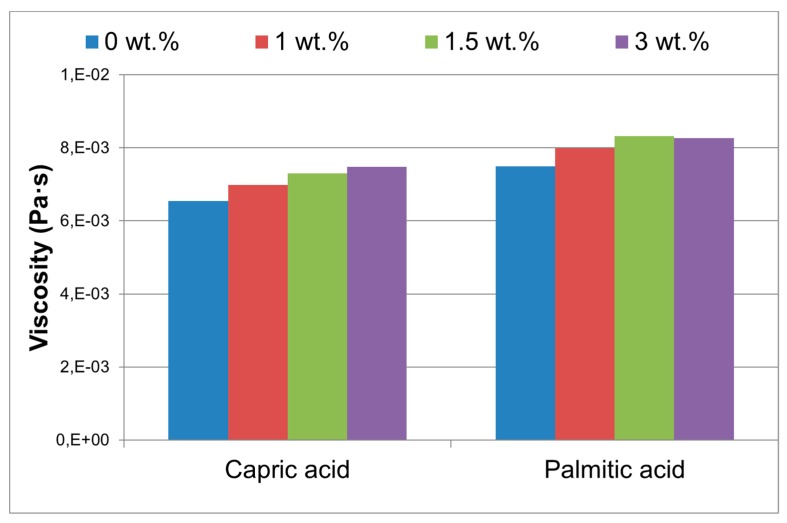
Viscosity of the NEPCM vs. the amount of CuO nanoparticles (wt.%).

**Viscosity:**Figure 4 shows the viscosity of the capric acid samples under study. There is a slight increment of the viscosity with the nanoparticles content, as expected. This fact will promote the NEPCM stability in terms of nanoparticles sedimentation.

The increment is gradual, but the maximum step is observed from 0.0 wt.% to 1.0 wt.% nanoparticles content.

**Sedimentation velocity:** The sedimentation velocity was calculated based on the results shown in Figure 4 and the density values provided in the technical data sheet for the fatty acids, and the theoretical value for CuO and following the Stokes law (Equation (1)). Sedimentation velocity results are listed in Table 1. 

Capric acid samples decrease slightly the sedimentation rate *V* when increasing the nanoparticles percentage and this effect is the same for the palmitic acid samples. Therefore, the nanoparticle sedimentation will need more time with higher nanoparticle content in the studied range (0–3 wt.%). Capric acid samples present a more similar density to the nanoparticles, and this fact makes the sedimentation time higher [21].

**Figure 5:**
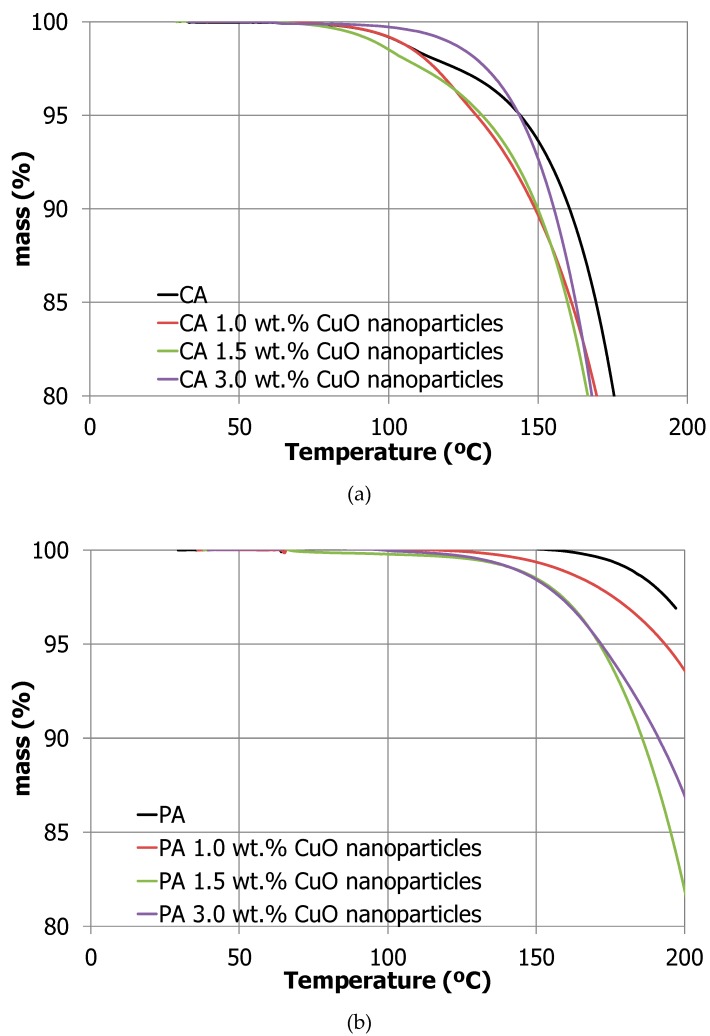
Thermogravimetric analyses (TGA) results of the NEPCM studied: (**a**) Capric acid samples; (**b**) Palmitic acid samples.

**Thermal stability:** TGA results shown in Figure 5 present information related to the maximum working temperature and the degradation temperature of the samples under study. 

By observing the results all samples under study follow a similar coherent decomposition procedure. As expected, palmitic acid samples are more stable starting their decomposition slightly above 185 °C, while capric acid samples start their decomposition around 108 °C. 

The presence of nanoparticles catalyzes the fatty acid degradation since the maximum working temperature (see Table 2) decrease around 30 °C when the samples have nanoparticles in the palmitic acid samples and this effect is around 10 °C for capric acid samples. Since the decomposition of both NEPCM starts at 185 °C and 108 °C, respectively, authors confirm that the PCM and the NEPCM under development are stable within the building’s applications temperature range.

**Figure 6:**
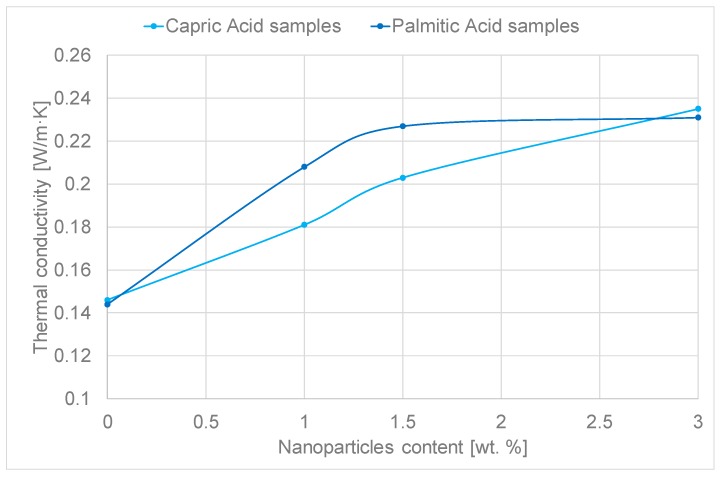
Effective thermal conductivity results vs. nanoparticles content.

**Effective thermal conductivity:** The studied samples were melted before the *κ* measurements. The measurements were performed at 40 °C (capric acid) and 70 °C (palmitic acid) and each *κ* measurement was repeated 6 times. The *κ* results are depicted in Figure 6. 

Capric acid samples increase their thermal conductivity with the nanoparticles increment. Therefore, the higher the nanoparticles content in the NEPCM the higher the effective thermal conductivity within the nanoparticle content range studied.

However, palmitic acid samples increase their thermal conductivity till 1.5 wt.% of nanoparticles up to 0.23 W/m·K and this value is stagnated independently of the nanoparticles content in NEPC M.

The increments related to the addition of nanoparticles to the capric acid reach 44% when addition 3.0 wt.% nanoparticles and in the case of palmitic acid reach 55% increment when addition 1.5 wt.% nanoparticles. These increments are slightly higher than those demonstrated using Fe_2_O_3_ nanoparticles (about 30% increment) [17] and Cu nanoparticles [22] in organic PCMs (around 20%).

**Figure 7:**
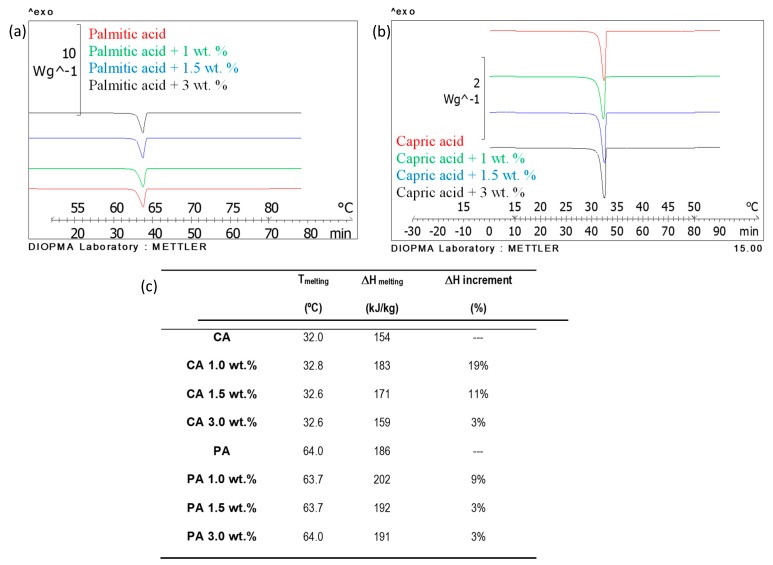
Differential scanning calorimeters (DSC) results of NEPCM manufactured in this study: (**a**) DSC results of palmitic acid samples, (**b**) DSC results of capric acid samples, (**c**) DSC results summary.

**Latent heat store:** DSC results are shown in Figure 7. The melting temperatures of the NEPCM measured are coherent with the fatty acid without nanoparticles. 

All samples under study present very high thermal storage capacity. Indeed, samples containing 1.0 wt.% nanoparticles present the maximum ΔH increment (19%) in capric acid case and 9% in palmitic acid case. These results were shown before by the results published by Sahan and Paksoy where they reported increments up to 27.3% of phase change enthalpy when adding nanomagnetite particles [23]. The melting temperature results are in concordance depending on the acid used to each sample under study. 

## 3. Materials and Methodology

### 3.1. Samples Preparation

The study presented in this paper focused on bio-based PCM which have a low environmental impact [24] and a better fire reaction behavior [25]. In particular, the bio-based PCM used in this work were decanoic acid (Sigma Aldrich) also known as capric acid (C_10_H_20_O_2_) with ≥98% purity and hexadecanoic acid (Sigma Aldrich) also known as palmitic acid (C_16_H_32_O_2_) with ≥98% purity. 

**Figure 8:**
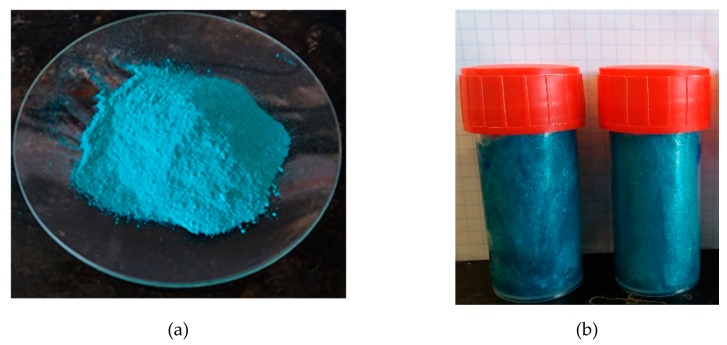
(**a**) Synthetized copper (II) oxide (CuO) nanoparticles; (**b**) nano-enhanced phase change materials (NEPCM) both prepared in the laboratory of the University of Barcelona.

The nanoparticles used in this study were synthetized in University of Barcelona labs (see Figure 8). 

Copper (II) oxide (CuO) nanoparticles were synthesized via a coprecipitation method by using copper chloride (CuCl_2_·2H_2_O, Panreac) as the metal precursor, octanoic acid (C_8_H_16_O_2_, Scharlab) and sodium hydroxide (NaOH, VWR Chemicals). All these chemicals were used as-received without any further purification. C_8_H_16_O_2_ worked as a surfactant agent for the two main purposes: (i) avoid particle growth and (ii) improve the dispersion of nanoparticles in the medium. 

A 0.1 M copper chloride solution was prepared by mixing CuCl_2_·2H_2_O with distilled water and stirring until complete dissolution. Then, a certain amount of C_8_H_16_O_2_ (1 mL/1g CuO) was added while stirring, and later a 1 M solution of NaOH was slowly added dropwise until a pH close to 10 was reached. The resultant solution was heated up to 80 °C and stirred for 1 hour and then cooled to room temperature. In order to get a material free from Na^+^ and Cl^−^ impurities, the sample was washed with a mixture of water and ethanol (50% *v*/*v*). To eliminate the supernatant liquid, the mixture was centrifuged during 10 minutes at 3000 rpm and the liquid decanted. This process was repeated three times. Then, neither further solvents nor temperature—based processes were used in order to avoid both, remove the surfactant and particle growth. Finally, the sample was dried at 105 °C for 7 hours and ground into powder with an agate mortar.

The preparation of NEPCM was performed using the two-step method [21,26]. This method consists on stirring the PCM (melted fatty acid) and adding the nanoparticles in order to avoid the nanoparticles agglomeration. After the direct mixture, an ultrasound device was used for 15 minutes to improve the nanofluid stabilization as well as the nanoparticles dispersion within the fluid.

Samples of each fatty acid were prepared and several nanoparticles percentage were added to each sample (0 wt.%, 1 wt.%, 1.5 wt.% and 3 wt.%) based on previous studies [27]. All samples prepared and under study are listed in Table 3. 

### 3.2. Methodology

#### 3.2.1. Nanoparticles Characterization

**Sample size**: Scanning electron microscopy (SEM) was used to evaluate the morphology of the synthetized nanoparticles and to measure their particle size. The instrument used was high resolution field emission scanning electron microscopy (FE-SEM) and the images were obtained by secondary electrons detector.

**Composition:** X-ray diffraction (XRD) was used to determine the nanoparticles composition after the synthesis process followed by the authors (see Section 3.1). The instrument used was XRD from Philips MRD (Amsterdam, Netherlands).

#### 3.2.2. NEPCM Characterization

**Composition:** The composition and the structure characterization of the NEPCM was performed by Fourier transformed infrared (FT-IR) spectroscopy, which is based on the chemical bond vibration when an infrared wave was applied to the sample under study. Each FT-IR peak is characteristic of each bond vibration mode and provides a fingerprint spectrum of functional groups and determines the major components of the substance. A Spectrum Two™ from Perkin Elmer (Waltham, MA, USA) was used to perform the FT-IR analyses coupled to an attenuated total reflectance (ATR) which allow the direct sample FT-IR measurements. 

**Viscosity:** The viscosity of the NEPCM was measured by means of a rheometer (Brookfield RST-CPS) (Middleboro, MA, USA) of both bio-based PCM doped with different nanoparticle percentages in liquid phase at 35 °C in the case of capric acid samples and 75 °C for palmitic acid samples. The measurement system was a cone plate RCT-50-1 and 500–5000 s^−1^ share rate. The viscosity results were also used to calculate the particles sedimentation following the Stokes law (Equarion (1)) [21].

**Density:** The bulk density of the NEPCM in liquid state was measured at our lab by measuring the mass of a contained known volume. The accuracy of the measurements is ±0.1 cm^3^.

**Sedimentation velocity:** The nanoparticles size was used to calculate the particles precipitation ratio following the Equation (1) (Stokes law [21]) by using the results obtained from the viscosity, density measurements, and SEM results.

(1) $$V=\frac{2\cdot a^{2}\left(\rho_{np}-\rho_{bf}\right)\cdot g}{9\cdot \eta }$$

where a is the particle radio, $\rho_{np}$ and $\rho_{bf}$ are the nanoparticles density and the NEPCM density, respectively, and η is the viscosity of the NEPCM, *V* is the sedimentation velocity, and g the gravitational acceleration.

The Stoke law describes how the nanoparticles within a fluid tend to agglomerate due to the Van der Waals forces and this behavior promotes gradual nanoparticle sedimentation. 

Notice that the sedimentation ratio decreases when the nanoparticles are smaller, the density of nanoparticles and fluids are similar, and the viscosity of the NEPCM is high.

Thermal stability of the NEPCM was measured by thermogravimetric analyses (TGA) in order to determine the maximum working temperature of the NEPCM under study. The equipment used was a TA Instrument simultaneous TGA/differential scanning calorimeters (DSC) (DSTQ600, New Castle, DE, USA) which has a balance sensitivity of 0.1 µg. TGA analyses were performed between 25 °C and 300 °C under 80 mL/min synthetic air flow and 10 K/min heating rate. The results will outline the maximum working temperature. The maximum working temperature was defined as the upper temperature limit where the sample was able to work without degradation (less than 1.5 wt.% mass loss).

**Effective thermal conductivity:** The technique used to measure the *κ* was the hot wire which is based on passing an electric pulse through a wire and due to the Joule effect the wire is heated. The system registers the temperature changes over time. The sensor used was KS-1 and it has 10% error within the range of 0.02–2 W/(m·K). The samples are measured in liquid phase at 40 °C for capric acid samples and 75 °C for palmitic acid samples. The equipment used to perform the *κ* measurements was a KD2 PRO (Decagon Devices Inc).

**Latent heat stored:** PCM store thermal energy from the change of state from solid to liquid those materials present when a temperature gradient is applied This heat is known as latent heat and DSC are the most powerful instruments to measure the thermophysical properties of a substance. The equipment used was a DSC 822e from Mettler Toledo. The measurements were performed following the methodology described by Miró et al. [28], between ±15 °C from the melting point, under 0.5 K/min heating rate and 50 mL/min N_2_ flow. The experimental error of phase change enthalpy is ±1 kJ/kg·K. The phase change temperature of all samples is also measured based on the same method here described. In this case, the error of phase change temperature is ±0.1 °C.

## 4. Conclusions

CuO nanoparticles were successfully prepared by coprecipitation and characterized by XRD and SEM. Afterwards, both fatty acids were enhanced in terms of TES when up to a 3.0 wt.% of CuO nanoparticles were added following the two-step’s method.

Capric and palmitic acid NEPCM compositions, and both themophysical and rheological properties were characterized. Whereas both NEPCM viscosities increased with nanoparticle content, the maximum increase is 7% for the 1.0 wt.% CuO nanoparticle content. This slightly higher viscosity can draw out the nanoparticle sedimentation process. Sedimentation rate was calculated using values from experimental results obtained in SEM, viscosity and density test. A slower sedimentation rate was measured for higher nanoparticles contents. Experimental results showed noteworthy increments in NEPCM thermal conductivity. In case of palmitic NEPCM the thermal conductivity stagnated after 1.5 wt.% while in capric NEPCM, the maximum increment achieved (55%) was measured at 3.0 wt.% CuO nanoparticle content. Latent heat storage capacity, on the other hand, showed slighter increments. The melting enthalpies of palmitic NEPCM samples had a maximum increase of 9% while capric NEPCM showed an enhancement of 19%. As FT-IR results demonstrated the composition of both fatty acid PCM does not change substantially after the CuO nanoparticles addition and sonication. Moreover, TGA analysis proves the NEPCM thermal stability within the building application temperature range. 

## Figures and Tables

**Table 1:** Sedimentation velocity results.

	α (m)	ρ_np_ (kg/m^3^)	ρ_bf_ (kg/m^3^)	η (kg/m·s)	S (m/s)
CA 1.0 wt.%	4.50 × 10^−8^	6300	893	6.99 × 10^−3^	3.41 × 10^−9^
CA 1.5 wt.%	4.50 × 10^−8^	6300	893	7.30 × 10^−3^	3.27 × 10^−9^
CA 3.0 wt.%	4.50 × 10^−8^	6300	893	7.48 × 10^−3^	3.19 × 10^−9^
PA 1.0 wt.%	4.50 × 10^−8^	6300	852	7.99 × 10^−3^	2.99 × 10^−9^
PA 1.5 wt.%	4.50 × 10^−8^	6300	852	8.24 × 10^−3^	2.90 × 10^−9^
PA 3.0 wt.%	4.50 × 10^−8^	6300	852	8.26 × 10^−3^	2.89 × 10^−9^

**Table 2:** Maximum working temperature of samples under study.

	CA	CA 1.0 wt.%	CA 1.5 wt.%	CA 3.0 wt.%	PA	PA 1.0 wt.%	PA 1.5 wt.%	PA 3.0 wt.%
**Maximum Working Temperature (°C)**	108.7	108.2	100.1	98.5	186.5	165.1	150.0	149.2

**Table 3:** List of samples under study.

Samples	Capric Acid	Palmitic Acid	Nano CuO Particles
CA	100.0 wt.%	---	---
CA 1.0 wt.%	99.0 wt.%	---	1.0 wt.%
CA 1.5 wt.%	98.5 wt.%	---	1.5 wt.%
CA 3.0 wt.%	97.0 wt.%	---	3.0 wt.%
PA	---	100.0 wt.%	---
PA 1.0 wt.%	---	99.0 wt.%	1.0 wt.%
PA 1.5 wt.%	---	98.5 wt.%	1.5 wt.%
PA 3.0 wt.%	---	97.0 wt.%	3.0 wt.%
